# HMGA1 Promotes Macrophage Recruitment via Activation of NF-*κ*B-CCL2 Signaling in Hepatocellular Carcinoma

**DOI:** 10.1155/2022/4727198

**Published:** 2022-06-22

**Authors:** Junming Chen, Kang Ji, Lingyan Gu, Yu Fang, Ming Pan, Shuxia Tian

**Affiliations:** Department of TCM, Minhang Hospital, Fudan University, TCM Building, No. 170, Xinsong Road, Minhang District, Shanghai, China

## Abstract

**Background:**

Tumor-associated macrophages (TAMs) are known to generate an immune-suppressive tumor microenvironment (TME) and promote tumor progression. Hepatocellular carcinoma (HCC) is a devastating disease that evolves in the background of chronic inflammatory liver damage. In this study, we aimed to uncover the mechanism by which HCC cells recruit macrophages into the TME.

**Methods:**

Bioinformatic analysis was performed to identify differentially expressed genes related to macrophage infiltration. An orthotopic HCC xenograft model was used to determine the role of macrophages in HCC tumor growth. Clodronate liposomes were used to delete macrophages. Western blotting analysis, quantitative real-time PCR, and enzyme-linked immunosorbent assay were performed to determine the underlying mechanisms.

**Results:**

The high mobility group A1 (HMGA1) gene was identified as a putative modulator of macrophage infiltration in HCC. Deletion of macrophages with clodronate liposomes significantly abrogated the tumor-promoting effects of HMGA1 on HCC growth. Mechanistically, HMGA1 can regulate the expression of C-C Motif Chemokine Ligand 2 (CCL2), also referred to as monocyte chemoattractant protein 1 (MCP1), which is responsible for macrophage recruitment. Moreover, NF-*κ*B was required for HMGA1-mediated CCL2 expression. Pharmacological or genetic inhibition of NF-*κ*B largely blocked CCL2 levels in HMGA1-overexpressing HCC cells.

**Conclusions:**

This study reveals HMGA1 as a crucial regulator of macrophage recruitment by activating NF-*κ*B-CCL2 signaling, proves that HMGA1-induced HCC aggressiveness dependents on the macrophage, and provide an attractive target for therapeutic interventions in HCC.

## 1. Introduction

Macrophages play critical roles in development, homeostasis, tissue repair, and innate and adaptive immunity [1]. Under pathophysiological conditions, macrophages exhibit phenotypic heterogeneity and functional diversity [2]. Tumor-associated macrophages (TAMs) are the most abundant tumor-infiltrating immune cell types with the tumor microenvironment (TME) and are generally categorized into two groups, the classically activated M1 macrophages and the alternatively activated M2 macrophages. M1 macrophages typically exert antitumor roles, while M2 macrophages suppress T cell-mediated antitumor immune response [3]. It is widely reported that TAMs can promote tumor growth, invasion, metastasis, immune-suppressive TME remodeling, and drug resistance [4–6]. Thus, a better understanding of the molecular mechanism by which TAMs promote tumor progression would enable the development of macrophage-targeting immunotherapies.

Hepatocellular carcinoma (HCC) is a common malignancy worldwide, and it has diverse etiologies with multiple mechanisms, especially a background of chronic liver disease. Unfortunately, most HCC patients are diagnosed at advanced stages and have few treatment options, and clinical outcome is extremely poor [7, 8]. Previously, accumulated evidence has shown that TAMs are crucial TME components that are essential for HCC development and represent a promising option for disrupting the pathogenesis of HCC [9–13]. For example, endothelial cells induce immunosuppressive macrophages in HCC and tumor-derived adenosine contributes to macrophage proliferation in HCC [14, 15]. However, the key players involved in the process of macrophage recruitment within the HCC TME remain areas of active investigation.

High mobility group (HMG) proteins are small nuclear proteins with high mobility. Three families of HMG proteins have been identified: HMGA, HMGB, and HMGN. All HMG proteins contain an acidic carboxyl terminus and modulate chromatin structure [16]. HMGA1 is well documented chromatin-associated protein and is known to participate in a myriad of cellular processes including transcriptional regulation, embryonic development, cell cycle progression, DNA damage response, cellular senescence, and mitochondrial function [17, 18]. Notably, HMGA1 is also reported to be an oncogene in diverse cancer types [17, 19–23]. For instance, HMGA1 is highly expressed in breast cancer and contributes to angiogenesis via promoting the nuclear localization and transcriptional activity of FOXM1 [24]. HMGA1 can also promote cancer stemness and epithelial-mesenchymal transition of perihilar cholangiocarcinoma via modulation of c-Myc [25]. In HCC, HMGA1 has been reported to promote tumor growth and metastasis and is a potential prognostic factor [26, 27]. However, limited is known about the link between HMGA1 and TAMs in HCC.

In this study, we identified that HMGA1 acts as an important regulator of macrophage infiltration in HCC. Deletion of macrophage in HCC tissues largely blocked the tumor-promoting effects of HMGA1 on HCC tumor growth. Furthermore, CCL2 was revealed to be a downstream target of HMGA1 to mediate macrophage recruitment. A further mechanism study showed that HMGA1 recruits the inflammatory transcriptional factor NF-*κ*B to induce CCL2 expression in HCC.

## 2. Materials and Methods

### 2.1. Bioinformatic Analysis

The TIMER database (https://cistrome.shinyapps.io/timer/) [28] was used to investigate the expression pattern of HMGA1 across human cancers, the prognostic value of HMGA1 in HCC, and the association between HMGA1 and immune cells. For the Kaplan-Meier curve analysis, the log-rank test was used. The online website Gene Expression Profiling Interactive Analysis (GEPIA) database (http://gepia.cancer-pku.cn/index.html) was employed to determine the correlation between HMGA1 expression and macrophage gene signatures, the correlation coefficient was detected by the Spearman method [29]. The M2 macrophage score in HCC samples was generated according to the CIBERSORT method.

### 2.2. Cell Culture and Reagents

The human liver cancer cell lines (HepG2, Huh7, Hep3B, SNU-423, HCC-LM3, MHCC-97H, SK-Hep1, and SMMC-7721) and two human immortalized normal liver cell lines (LO2 and THLE-2) were obtained from the Institute of Biochemistry and Cell Biology at the Chinese Academy of Sciences (Shanghai, China) or American Type Culture Collection (ATCC) and supplemented with Dulbecco's modified Eagle's medium (DMEM; Gibco, USA) mixed with 10% fetal bovine serum (FBS; Gibco, USA) and 1% antibiotics (penicillin and streptomycin) at 37°C in a humidified incubator containing 5% CO_2_. All the cell lines have been tested for contamination before cell experiments. The I*κ*B*α* phosphorylation inhibitor Bay11-7082 was purchased from Selleck (S2913, Shanghai, China) and dissolved in dimethyl sulfoxide (DMSO; as a vehicle).

### 2.3. Cell Transfection

The specific siRNA against HMGA1 and negative control (NC-siRNA) was synthesized by GenePharma (Shanghai, China). Indicated HCC cells were cultured in 6-well plates and were transiently transfected with si-HMGA1 or NC-siRNA by using Lipofectamine 2000 reagent (Invitrogen, Carlsbad, CA, USA) according to the manufacturer's protocols. The siRNAs used in this study were as follows: HMGA1-siRNA1, 5′-GUGCCAACACCUAAGAGACCUTT-3′; HMGA1-siRNA2, 5′-GCAGGAAAAGGACGGCACUTT-3′; and the negative control NC-siRNA: 5′-UUGUACUACACAAAAGUACUG-3′. For HMGA1 overexpression in SUN-423 cells, the full-length HMGA1 cDNAs were cloned into the Lentiviral vectors pcDNA3.1 (+). Similarly, the constructs were transfected using Lipofectamine 2000 according to the product manual. The stable cells were selected using G418 after viral infection.

### 2.4. Western Blotting

Total protein was extracted from liver cancer cells with RIPA Lysis Buffer [50 mM Tris-HCl (pH 7.4), 0.15 M NaCl, 1% NP-40, 0.25% Na-deoxydiolate, 1 mM EDTA] containing protease inhibitors (1 mM phenylmethylsulfonyl fluoride, 1 *μ*g/ml aprotinin, 1 mM Na_3_VO_4_, 1 Mm NaF). The protein concentration was determined with a Pierce BCA protein assay kit (Thermo Fisher Scientific, Inc., USA). Then, 20-40 *μ*g protein was separated by 8-12% sodium dodecyl sulfate-polyacrylamide gel electrophoresis (SDS-PAGE) and transferred onto polyvinylidene fluoride (PVDF) membranes, followed by blocking with 10% nonfat milk/TBST at room temperature for 1 h and probing with the following primary antibodies at 4°C overnight. The following antibodies were used in this study, anti-HMGA1 (1 : 1,000; ab129153; Abcam), anti-p-P65 (1 : 1,000; #3033; Cell Signaling Technology), and anti-P65 (1 : 1,000; #8242; Cell Signaling Technology). On the second day, the membranes were probed with a secondary antibody and the bands were detected by enhanced chemiluminescence reagents (Pierce, USA). *β*-Actin (1 : 1,000; ab8227; Abcam) was used as a protein loading control and total protein levels were normalized to *β*-actin.

### 2.5. Real-Time Quantitative PCR

Total RNA was extracted from indicated cell lines using RNAiso Plus reagent (TaKaRa, Japan) according to the manufacturer's instructions. Complementary DNA (cDNA) cDNA synthesis was conducted by PrimeScript RT Reagent Kit with gDNA Eraser (TaKaRa, Japan). Real-time PCR was performed using SYBR Premix Ex Taq (TaKaRa, Japan) in ABI 7500 system (Applied Biosystems, USA) according to the manufacturer's protocol. The primer sequences used in this study were shown as follows: HMGA1 forward 5′-AGCGAAGTGCCAACACCTAAG-3′, HMGA1 reverse 5′-TGGTGGTTTTCCGGGTCTTG-3′; CCL2 forward 5′-CAGCCAGATGCAATCAATGCC-3′, CCL2 reverse 5′-TGGAATCCTGAACCCACTTCT-3′; GAPDH forward 5′-CTGGGCTACACTGAGCACC-3′, GAPDH reverse 5′-AAGTGGTCGTTGAGGGCAATG-3′. Relative mRNA expression was normalized to GAPDH according to the 2^−ΔΔCt^ method.

### 2.6. Immunofluorescence Analysis

After the removal of cell culture medium, ov-vector and ov-HMGA1 SNU-423 cells were washed with cold PBS, fixed with 4% paraformaldehyde at room temperature for 15 min, and permeabilized with 0.1% Triton X100 for 5 min. Then, the cell samples were blocked with 5% FBS and stained by primary antibody solution (1 : 200; ab129153; Abcam) at 4°C for overnight. On the next day, the cell samples were incubated with secondary antibody at room temperature for 1 h and counterstained with diamidino phenylindole (DAPI) for 10 min. Finally, the HMGA1 signals were analyzed by confocal fluorescence microscopy (Nikon, Tokyo, Japan).

### 2.7. Immunohistochemistry (IHC)

Paraffin-embedded sections of lung and liver tissues were analyzed to determine F4/80-positive macrophages. After deparaffinization, lung and liver tissue slides were rehydrated and subjected to antigen retrieval by microwaving in 0.01 mol/L sodium citrate (pH 6.0) for 10 min. After blocking endogenous peroxidase, the tissue sections were incubated with antibodies against F4/80 (1 : 200; #70076; Cell Signaling Technology) 4°C overnight. Immunostaining was performed using DAB Kit (#8059; Cell Signaling Technology) according to the manufacturer's instructions. Subsequently, sections were counterstained with haematoxylin. Finally, positive staining in the tissue sections was evaluated by an expert pathologist.

### 2.8. *In Vivo* Animal Study

An orthotopic HCC xenograft model was generated to investigate the *in vivo* effects of HMGA1. Firstly, C57BL/6J mice were subjected to macrophage depletion assay. Twenty mice were injected intraperitoneally with clodronate liposomes or PBS liposomes twice a week for 4 weeks. Subsequently, 5 × 10^5^ ov-vector or ov-HMGA1 SNU-423 cells were injected orthotopically into the liver of C57BL/6J mice. Two weeks later, bioluminescent imaging analysis was performed to analyze the HCC tumor burden using an IVIS Spectrum system (PerkinElmer, Waltham, MA, USA). All mice were housed in laminar flow cabinets with free access to food and water. All animal experimental procedures were performed in accordance with the animal protocols and regulations approved by the Laboratory Animal Center of Minhang Hospital, Fudan University.

### 2.9. Enzyme-Linked Immunosorbent Assay (ELISA)

The secreted level of CCL2 in HCC cell culture supernatants was analyzed by ELISA. Briefly, indicated HCC cells were cultured with FBS-free DMEM for 24 h. Then, the cell supernatants were collected, cleared by centrifugation, and used for ELISA experiment immediately. The commercial Human CCL2/MCP-1 Quantikine ELISA Kit (Catalog # DCP00, R&D Systems) was used to determine CCL2 in the cell supernatants according to the manufacturer's instructions. The final concentration of CCL2 was normalized to the total cell number.

### 2.10. Chemotaxis Assay

Monocytes derived from peripheral blood mononuclear cells (PBMCs) were used for chemotaxis assay. Indicated HCC cells were cultured with FBS-free DMEM for 24 h and conditioned medium was collected. PBMCs were isolated from the venous blood of healthy donors by density gradient centrifugation. The Transwell chambers were used to determine the migratory ability of monocytes. Briefly, 2 × 10^4^ monocytes were plated in the upper compartment of Transwell chambers, and the lower chamber contained RPMI 1640 medium with indicated conditioned medium or recombinant CCL20 protein (Catalog #279-MC, R&D Systems, USA). Twenty-four hours later, the migrated monocytes were fixed with ethanol for 30 min and stained with 0.5% crystal violet for 30 min. Cells were counted in six randomly selected fields under a microscope. All experiments were performed in triplicate.

### 2.11. Statistical Analysis

Statistical analysis was performed by SPSS 22.0 (IBM, NY, USA) or Prism 5 (GraphPad Software, San Diego, CA, USA). Values were expressed as the mean ± standard deviation (SD). Statistical analysis was performed using Student's *t*-test for pairwise comparison, one-way analysis of variance test for multiple group comparisons. The correlation of gene expression was evaluated by Spearman's correlation. A *P* value <0.05 was considered statistically significant; ^∗^*P* < 0.05; ^∗∗^*P* < 0.01; ^∗∗∗^*P* < 0.001.

## 3. Results

### 3.1. HMGA1 Expression Is Associated with Macrophage Infiltration in HCC

**Figure 1:**
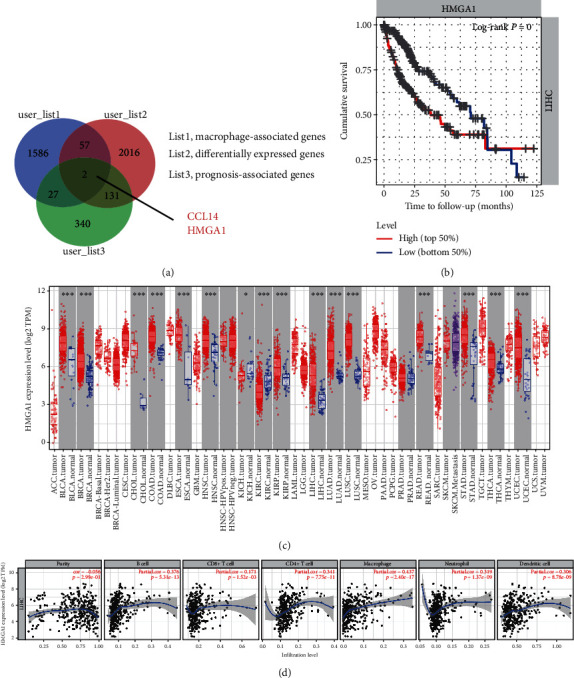
HMGA1 expression is associated with macrophage infiltration in HCC. (a) Venn diagram showed the macrophage-associated genes, differentially expressed genes (DEGs), and prognosis-associated genes in HCC. (b) The Kaplan-Meier curve showed the prognostic value of HMGA1 in HCC; data were obtained from the TCGA cohort and the median value of HMGA1 was set as a cutoff; high group (*n* = 182) and low group (*n* = 182). (c) The pan-cancer expression pattern of HMGA1 in tumor tissue and corresponding nontumor tissues was acquired from the TIMER database (https://cistrome.shinyapps.io/timer/). ACC = adrenocortical carcinoma; BLCA = bladder urothelial carcinoma; BRCA = breast invasive carcinoma; CESC = cervical squamous cell carcinoma and endocervical adenocarcinoma; CHOL = cholangiocarcinoma; COAD = colon adenocarcinoma; DLBC = lymphoid neoplasm diffuse large B-cell lymphoma; ESCA = esophageal carcinoma; GBM = glioblastoma multiforme; HNSC = head and neck squamous cell carcinoma; KICH = kidney chromophobe; KIRC = kidney renal clear cell carcinoma; KIRP = kidney renal papillary cell carcinoma; LAML = acute myeloid leukemia; LGG = brain lower grade glioma; LIHC = liver hepatocellular carcinoma; LUAD = lung adenocarcinoma; LUSC = lung squamous cell carcinoma; MESO = mesothelioma; OV = ovarian serous cystadenocarcinoma; PAAD = pancreatic adenocarcinoma; PCPG = pheochromocytoma and paraganglioma; PRAD = prostate adenocarcinoma; READ = rectum adenocarcinoma; SARC = sarcoma; SKCM = skin cutaneous melanoma; STAD = stomach adenocarcinoma; TGCT = testicular germ cell tumors; THCA = thyroid carcinoma; THYM = thymoma; UCEC = uterine corpus endometrial carcinoma; UCS = uterine carcinosarcoma; UVM = uveal melanoma. (d) The association between HMGA1 expression and immune components (B cell, CD8+ T cell, CD4+ T cell, macrophage, neutrophil, and dendritic cell) in HCC.

To investigate the key player involved in the recruitment of macrophages in HCC, we acquired the score of M2 macrophage by adopting the CIBERSORT method [30] and identified differentially expressed genes using a dichotomous analysis. In this case, 1672 genes associated with macrophage infiltration were identified. To narrow the targets, we further identified 2206 differentially expressed genes in HCC tissues and 500 genes related to the patient's prognosis. By merging genes in these three lists, 2 genes, named CCL14 and HMGA1, were found. Given that CCL14 is known to modulate macrophage function, we selected HMGA1 for further investigation (Figure 1(a)). In the TCGA cohort, higher HMGA1 expression predicted a poor clinical outcome in HCC patients as evidenced by the Kaplan-Meier curve analysis (Figure 1(b)). Consistent with the previous reports, HMGA1 was frequently overexpressed in human cancers, including liver hepatocellular carcinoma (LIHC) (Figure 1(c)). To confirm the link between HMGA1 and macrophage, we performed correlation analysis using the online database TIMER (https://cistrome.shinyapps.io/timer/). As expected, HMGA1 expression had a higher correlation coefficient with macrophage compared with other immune cells (B cell, CD8+ T cell, CD4+ T cell, neutrophil, and dendritic cell) in HCC (Figure 1(d)). Thus, these findings suggest that HMGA1 might act as a modulator of macrophage recruitment in HCC.

### 3.2. Depletion of Macrophage Mitigates HMGA1-Induced Tumor Growth in HCC

**Figure 2:**
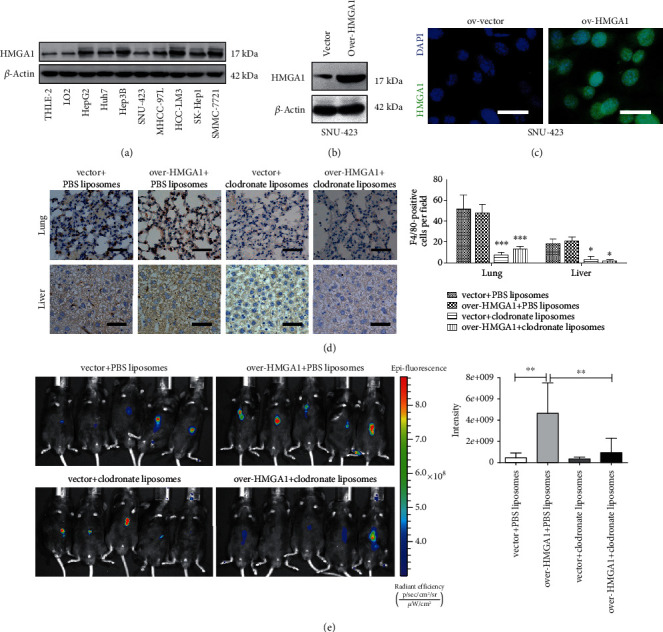
Depletion of macrophage mitigates HMGA1-induced tumor growth in HCC. (a) Western blotting analysis showed the protein level of HMGA1 in eight liver cancer cell lines and two normal control cell lines (LO2 and THLE-2). (b) SUN-423 cells were transfected with ov-HMGA1 or empty vector lentivirus, and Western blotting analysis showed the protein level of HMGA1 in ov-vector and ov-HMGA1 SUN-423 cells. (c) Immunofluorescence analysis showed the protein level and distribution of HMGA1 in ov-vector and ov-HMGA1 SUN-423 cells; scale bar: 10 *μ*m. (d) An orthotopic xenograft model was generated to determine the in vivo effect of HMGA1 in HCC; immunohistochemical analysis was performed to investigate the F4/80-positive cells in liver and lung tissues obtained from C57BL/6J mice treated with either clodronate liposomes or PBS liposomes; scale bar: 50 *μ*m. **(**e**)** In vivo imaging analysis of tumors from the ov-vector, ov-HMGA1, ov-vector plus macrophage deletion, and ov-HMGA1 plus macrophage deletion groups (*n* = 5 per group). The ANOVA followed by post hoc Tukey's multiple comparison test was used for group comparisons. ^∗^*P* < 0.05; ^∗∗^*P* < 0.01; ^∗∗∗^*P* < 0.001.

Previously, accumulated evidence has demonstrated that HMGA1 is overexpressed and plays diverse tumor-promoting effects in HCC and other cancer types [17]. Indeed, the Western blotting analysis showed that HMGA1 was highly expressed in HCC cell lines relative to two normal liver cell lines (LO2 and THLE-2) (Figure 2(a)). To determine whether the tumor-promoting effects of HMGA1 are associated with macrophage infiltration, we generated an orthotopic xenograft model by using overexpression strategies in SUN-423 cells, which present lower intrinsic HMGA1 protein expression. The protein levels of HMGA1 in ov-vector and ov-HMGA1 SUN-423 cells were verified by Western blotting analysis (Figure 2(b)) and immunofluorescence analysis (Figure 2(c)), respectively. Clodronate liposomes were used to deplete macrophages in the mouse tissues. Compared with phosphate-buffered saline (PBS) liposomes, clodronate liposomes significantly blocked macrophage infiltration in the lung and liver tissues as demonstrated by F4/80 staining (Figure 2(d)). As displayed by in vivo imaging analysis, HMGA1 overexpression remarkably increased tumor burden in immune-competent mice (Figure 2(e)). When macrophages were depleted, however, the tumor-promoting roles of HMGA1 were significantly blocked. Taken together, these data indicate that HMGA1 promotes tumor growth of HCC, at least, in part, dependent on macrophage infiltration.

### 3.3. CCL2 Is Required for HMGA1-Induced Macrophage Recruitment

**Figure 3:**
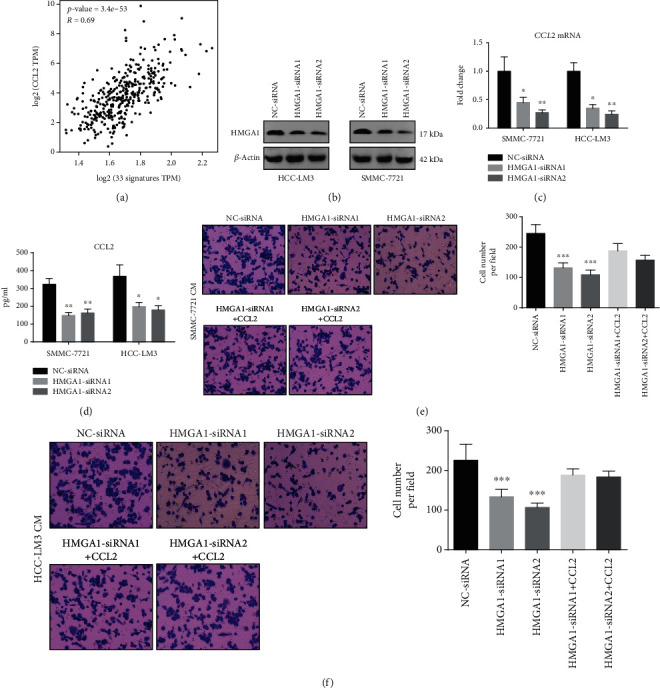
CCL2 is required for HMGA1-induced macrophage recruitment. (a) A gene signature consists of 33 genes (AIF1, CCL1, CCL14, CCL23, CCL26, CD300LB, CNR1, CNR2, EIF1, EIF4A1, FPR1, FPR2, FRAT2, GPR27, GPR77, RNASE2, MS4A2, BASP1, IGSF6, HK3, VNN1, FES, NPL, FZD2, FAM198B, HNMT, SLC15A3, CD4, TXNDC3, FRMD4A, CRYBB1, HRH1, and WNT5B) was used to define infiltrating macrophages, and the correlation between CCL2 and macrophage was investigated by Spearman's analysis. (b) HCC-LM3 and SMC-7721 cells were transfected with two specific siRNAs against HMGA1 or negative control (NC) siRNAs, and Western blotting analysis showed the protein level of HMGA1 in the HCC-LM3 and SMC-7721 cells. (c) Real-time qPCR analysis showed the mRNA level of CCL2 in the HCC-LM3 and SMC-7721 cells transfected with NC-siRNA or HMGA1-siRNA1/2. (d) Enzyme-linked immunosorbent assay (ELISA) showed the secreted level of CCL2 in the conditioned medium (CM) of HCC-LM3 and SMC-7721 cells transfected with NC-siRNA or HMGA1-siRNA1/2. (e and f) HCC-LM3 and SMC-7721 cells were treated with NC-siRNA or HMGA1-siRNA1/2 for 48 h, and CM was acquired and subjected for Transwell assay; the migratory ability of human monocytes isolated from PBMCs was evaluated after stimulation with indicated CM and recombinant human CCL2 protein for 12 h. The ANOVA followed by post hoc Tukey's multiple comparison test was used for group comparisons. ^∗^*P* < 0.05; ^∗∗^*P* < 0.01; ^∗∗∗^*P* < 0.001.

Next, we aimed to determine which chemokine is responsible for HMGA1-induced macrophage infiltration in HCC. It is known that CCL2 is a driver factor for macrophage recruitment in the tumor microenvironment [31]. CCL2-dependent macrophage recruitment is also widely reported in HCC [32]. By Spearman's correlation analysis with TCGA data, we revealed that CCL2 expression was positively associated with the macrophage infiltration in HCC (Figure 3(a)). Therefore, we tested the possible link between HMGA1 and CCL2. Using two specific siRNAs against HMGA1, we genetically silenced HMGA1 expression in two HCC cell lines (HCC-LM3 and SMC-7721) with higher HMGA1 expression. The knockdown efficiency of HMGA1 was confirmed by Western blotting analysis (Figure 3(b)). Real-time qPCR analysis showed the mRNA level of CCL2 in the HCC-LM3 and SMC-7721 cells was significantly downregulated by HMGA1 knockdown (Figure 3(c)). Consistently, the secreted level of CCL2 in the cell culture supernatants of HCC-LM3 and SMC-7721 cells was also reduced after HMGA1 knockdown (Figure 3(d)). Using Transwell assay, we investigated the chemotactic roles of conditioned medium (CM) from HCC-LM3 and SMC-7721 cells in human peripheral monocytes. As shown in Figures 3(e)–3(f), CM from HMGA1-siRNA1/2 HCC cells had a reduced chemotactic effect for human monocytes compared with CM from NC-siRNA HCC cells; notably, the addition of recombinant human CCL2 protein in the HMGA1-siRNA1/2 CM increased the migratory ability of human monocytes. Therefore, HMGA1 can induce CCL2 expression to recruit macrophages in HCC.

### 3.4. HMGA1 Regulates CCL2 Expression in an NF-*κ*B-Dependent Manner

**Figure 4:**
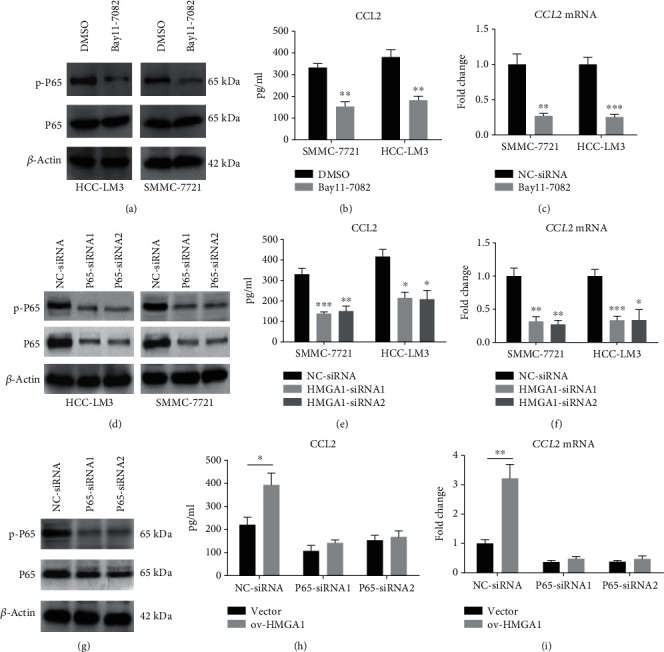
HMGA1 regulates CCL2 expression in an NF-*κ*B-dependent manner. (a) Western blotting analysis showed the total and phosphorylated level of P65 in HCC-LM3 and SMC-7721 cells after treatment with NF-*κ*B inhibitor Bay11-7082 (10 *μ*M). (b) ELISA analysis showed the secreted level of CCL2 in the conditioned medium (CM) of HCC-LM3 and SMC-7721 cells after treatment with 10 *μ*M Bay11-7082 for 24 h. (c) Real-time qPCR analysis showed the mRNA level of CCL2 in the HCC-LM3 and SMC-7721 cells after treatment with 10 *μ*M Bay11-7082 for 24 h. (d) Western blotting analysis showed the total and phosphorylated level of P65 in HCC-LM3 and SMC-7721 cells after treatment with specific siRNAs against P65. (e) ELISA analysis showed the secreted level of CCL2 in the conditioned medium (CM) of HCC-LM3 and SMC-7721 cells after treatment with specific P65 siRNAs for 48 h. (f) Real-time qPCR analysis showed the mRNA level of CCL2 in the HCC-LM3 and SMC-7721 cells after treatment with specific P65 siRNAs for 48 h. (g) Western blotting analysis showed the total and phosphorylated level of P65 in SNU-423 cells after treatment with specific siRNAs against P65. (h) ELISA analysis showed the secreted level of CCL2 in the conditioned medium (CM) of ov-vector and ov-HMGA1 SNU-423 cells after treatment with specific P65 siRNAs for 48 h. (i) Real-time qPCR analysis showed the mRNA level of CCL2 in the ov-vector and ov-HMGA1 SNU-423 cells after treatment with specific P65 siRNAs for 48 h. The ANOVA followed by post hoc Tukey's multiple comparison test or the Student's *t*-test was used for group comparisons. ^∗^*P* < 0.05; ^∗∗^*P* < 0.01; ^∗∗∗^*P* < 0.001.

**Figure 5:**
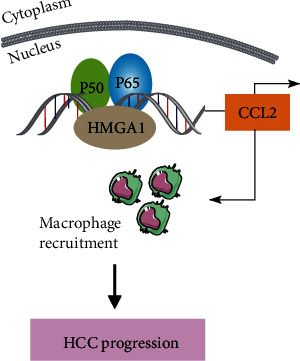
Mechanism model for HMGA1-mediated CCL2 expression in HCC. HMGA1 is located in the nucleus and functions together with NF-*κ*B to induce CCL2 expression. Furthermore, CCL2 recruits macrophages to the tumor microenvironment and leads to tumor progression.

Finally, we investigated the molecular mechanism by which HMGA1 increases CCL2 expression in HCC. Considering previous reports that HMGA1 and NF-*κ*B proteins (p50/p65) cooperate to induce inflammatory signaling cascades and the NF-*κ*B pathway is one of the main regulators of CCL2 expression [33], we inhibited this pathway with the I*κ*B*α* phosphorylation inhibitor Bay11-7082 and evaluated CCL2 expression (Figures 4(a)–4(c)). The phosphorylated level of P65 in HCC-LM3 and SMC-7721 cells was significantly downregulated after treatment with 10 *μ*M Bay11-7082 (Figure 4(a)). In line with decreased NF-*κ*B activity, the secreted and mRNA levels of CCL2 were reduced by Bay11-7082 treatment as demonstrated by ELISA analysis (Figure 4(b)) and real-time qPCR analysis (Figure 4(c)), respectively. Similarly, knockdown of P65 phenocopied the effects of Bay11-7082 on CCL2 expression in HCC-LM3 and SMC-7721 cells (Figures 4(d)–4(f)). Moreover, we analyzed the effect of HMGA1 on CCL2 expression in the presence or absence of P65 knockdown in SNU-423 cells. The results showed that HMGA1 overexpression increased the secreted and mRNA levels of CCL2, which can be blocked by P65 knockdown (Figures 4(g)–4(i)). Taken together, these data above indicate that HMGA1 could enhance NF-*κ*B function in the transcriptional regulation of CCL2 expression (Figure 5).

## 4. Discussion

The immune composition of the TME is of great importance in determining tumor progression or repression. TAMs are the major recruited immune cells in the TME and their phenotype status is detrimental to tumor growth and dissemination. Increased infiltration of TAMs is associated with a poor prognosis in most solid tumors. Importantly, targeting TAMs, such as inhibition of macrophage recruitment, reeducation of TAMs to “M1-like” mode, and monoclonal antibodies, efficiently destroys cancer cells [3]. Tumor cell-derived CCL2 is known to recruit macrophages, which are further reprogrammed to TAMs by milieu with the TME. Data from preclinical models showed that inhibition of CCL2-CCR2 signaling can reduce TAM infiltration and enhance the efficacy of immunotherapy [34, 35]. More interestingly, CCL2 is highly expressed in HCC and predicts a poor prognosis. Blockade of CCL2-CCR2 signaling inhibits TAM recruitment, attenuates HCC growth and metastasis, reduces postsurgical recurrence, and improves survival [32]. Here, we show that elevated HMGA1 leads to increased expression of CCL2 and subsequent infiltration of macrophages.

The expression level of HMGA1 is low or undetectable in normal tissues, while high levels of HMGA1 can be observed in primary or metastatic tumors from diverse tissues, indicating a putative role for HMGA1 in neoplastic transformation [17]. Previously, accumulated data pointed the regulatory roles of HMGA1 in cancer cell proliferation, invasion, metastasis, stemness, and drug resistance through multiple mechanisms [19, 25, 36, 37], including HCC. However, limited information is available about the link between HMGA1 and the tumor immune microenvironment. Previously, several inflammatory genes have been identified as target genes of HMGA1, such as inflammatory cytokine (IFN-*β*, IFN-*γ*, IL-4, IL-2, E-Selectin, P-Selectin, tumor necrosis factor-*β*, and CXCL1) and cytokine receptor genes (IL-2RA, CXCR3, and TCR*α*) [38]. In the present study, we for the first time showed that HMGA1 can induce CCL2 expression to recruit macrophages. Consistent with our observations, a recent report in HCC also found that the amount of M2 macrophages decreased when HMGA1 expression was high, whereas M0 macrophages increased [39]. Therefore, our findings provide new insight into HMGA1-mediated immune function and further broaden the oncogenic roles of HMGA1 in cancers. Notably, we cannot rule out the possibility of macrophage-independent roles of HMGA1 in HCC.

It has been reported that HMGA1 functions as an ancillary transcription factor that binds chromatin and recruits other transcription factors to DNA. Indeed, several studies have revealed that HMGA1 and NF-*κ*B can function together to regulate gene expression [16, 40]. Here, we also identified NF-*κ*B signaling as a molecular mechanism for translational regulation of CCL2 in HCC. Inhibition of the NF-*κ*B signaling or P65 knockdown compromised HMGA1-mediated CCL2 expression, suggesting that NF-*κ*B-mediated promoter activity is required for the role of HMGA1. However, global chromatin immunoprecipitation coupled with sequencing technology is encouraged to uncover HMGA1-dependent transcriptional networks in HCC. Given that HMGA1 is a chromatin-remodeling protein, possible epigenetic mechanisms for CCL2 expression are also present and warrant further investigation.

In conclusion, this study helps to unravel the complexity of HMGA1 function in HCC and provides insights into how HMGA1 affects macrophage infiltration in the TME of HCC. Moreover, our findings point to the HMGA1-NF-*κ*B-CCL2 signaling pathway that could serve as therapeutic targets in HCC and other human cancers with aberrant HMGA1 expression.

## Data Availability

The data used to support the findings of this study are available from the corresponding author upon request.
